# Long-term stability of clinically relevant chemistry analytes in pleural and peritoneal fluid

**DOI:** 10.11613/BM.2020.020701

**Published:** 2020-04-15

**Authors:** Lara Milevoj Kopcinovic, Marija Brcic, Alen Vrtaric, Adriana Unic, Marija Bozovic, Nora Nikolac Gabaj, Marijana Miler, Jelena Culej

**Affiliations:** Department of Clinical Chemistry, Sestre milosrdnice University Hospital Centre, Zagreb, Croatia

**Keywords:** stability, pleural effusion, peritoneal effusion, preanalytical phase

## Abstract

**Introduction:**

Our aim was to investigate the stability of clinically relevant analytes in pleural and peritoneal fluids stored in variable time periods and variable storage temperatures prior to analysis.

**Materials and methods:**

Baseline total proteins (TP), albumin (ALB), lactate dehydrogenase (LD), cholesterol (CHOL), triglycerides (TRIG), creatinine (CREA), urea, glucose and amylase (AMY) were measured using standard methods in residual samples from 29 pleural and 12 peritoneal fluids referred to our laboratory. Aliquots were stored for 6 hours at room temperature (RT); 3, 7, 14 and 30 days at - 20°C. At the end of each storage period, all analytes were re-measured. Deviations were calculated and compared to stability limits (SL).

**Results:**

Pleural fluid TP and CHOL did not differ in the observed storage periods (P = 0.265 and P = 0.170, respectively). Statistically significant differences were found for ALB, LD, TRIG, CREA, urea, glucose and AMY. Peritoneal fluid TP, ALB, TRIG, urea and AMY were not statistically different after storage, contrary to LD, CHOL, CREA and glucose. Deviations for TP, ALB, CHOL, TRIG, CREA, urea and AMY in all storage periods tested for both serous fluids were within the SL. Deviations exceeding SL were observed for LD and glucose when stored for 3 and 7 days at - 20°C, respectively.

**Conclusions:**

TP, ALB, CHOL, TRIG, CREA, urea and AMY are stable in serous samples stored up to 6 hours at RT and/or 30 days at - 20°C. Glucose is stable up to 6 hours at RT and 3 days at - 20°C. The stability of LD in is limited to 6 hours at RT.

## Introduction

Serum, plasma and urine (*i.e.* standard sample types) are the most frequent sample types analysed in the clinical laboratory. However, occasionally the analysis of pleural and peritoneal fluid (ascites) is also requested, especially when their aetiology is not evident from clinical signs and imaging studies (*1*, *2*).

For decades, various clinically relevant chemistry analytes have been used to help differentiate pleural and peritoneal effusions (*i.e.* serous effusions) and diagnose the cause of their accumulation. These have been determined using assays intended for standard sample types, making their analysis available and inexpensive. However, assays used in pleural and peritoneal fluid testing are not validated for this specific purpose (*i.e*. they lack manufacturer‘s analytical performance specifications for these sample types) (*3*-*5*). According to laboratory regulation authorities, the use of assays outside the manufacturer’s intended scope is considered a method modification (*6*-*9*). Thus, clinical laboratories are requested to review the manufacturer’s performance claims for standard fluids and validate their possible application to pleural and peritoneal fluid analysis (*5*, *7*, *9*, *10*). Serous body fluid validation is extensive, costly, challenging and requires careful planning. Additionally, serous fluid validation is hampered by the lack of commercially available matrix-matched quality control (QC) materials (*9*, *10*). This introduces the necessity to collect an appropriate volume/number of residual serous fluid samples to be utilized for validation experiments. Considering the (low) frequency of analysis of such samples in each clinical laboratory (*i.e.* their scarce availability), they need to be stored in appropriate storage conditions for extended time periods until analysis.

Stability of a measurand in a sample is a function of property variation over time in specific storage conditions (*11*). A multitude of studies are available in the literature exploring the stability of clinically relevant chemistry analytes in standard fluids like serum or plasma. However, although a number of studies pertaining to non-standard fluid validation (including pleural and peritoneal fluid) have been published in recent years, data specifically addressing the stability of analytes tested in pleural and peritoneal fluid samples are very limited (*4*, *12*-*14*). Nevertheless, as for standard fluids, stability is a critical preanalytical aspect of pleural and peritoneal fluid analysis. Thus, prior to initiating the serous fluids validation procedure, it needs to be considered in order to enable the appropriate use of the results obtained (*15*, *16*).

In the present study, our aim was to address this key component of pleural and peritoneal fluid validation and analysis; specifically, to investigate the stability of clinically relevant analytes in pleural and peritoneal effusions stored under different storage conditions prior to analysis. Our principal guiding thought was to obtain reliable and transferable stability results which might be used in future validation experiments of these off-label body fluids.

Since guidelines on how to perform a standardized stability study are lacking, we followed the checklist proposed by the Working Group for Preanalytical Phase (WG-PRE) of the European Federation for Clinical Chemistry and Laboratory Medicine (EFLM) and tried to implement all relevant information in our study (*17*).

## Materials and methods

This investigation was performed at the Department of Clinical Chemistry, Sestre milosrdnice University Hospital Centre, from September 2018 to January 2019 and approved by the institutional Ethics Committee. The investigation was conducted in accordance with the Helsinki Declaration. Since residual sample material (leftovers after routine analysis) was used, no informed consent was required (*18*).

A total of 44 consecutive pleural and peritoneal fluid samples (31 pleural and 13 peritoneal) were collected by thoracentesis/paracentesis on clinical wards in plain, white capped (no additive) tubes (Vacuette Tube Z No Additive, 3.5 mL, Ref. 454045, Greiner Bio-One GmbH, Kremsmünster, Austria) as part of routine clinical protocols from inpatients. After collection, samples were transported to the laboratory by authorized ward personnel. Upon receipt to the laboratory, samples were centrifuged for 10 minutes at 1800xg using a Rotofix 32A centrifuge (Hettich Zentrifugen, Tuttlingen, Germany). After completion of routine chemistry analysis, residual pleural and peritoneal fluid samples were obtained to be included in our stability study. Due to insufficient sample volume three samples were excluded at baseline, leaving a total of N = 41 (29 pleural and 12 peritoneal) samples for stability investigation. Baseline concentrations/activities for total proteins (TP), albumin (ALB), lactate dehydrogenase (LD), cholesterol (CHOL), triglycerides (TRIG), creatinine (CREA), urea, glucose and amylase (AMY) were measured within one hour of sample receipt to the laboratory, using the Abbott Architect c8000 (Abbott Laboratories, Abbott Park, USA) and corresponding proprietary reagents intended for use with serum (standard) samples, as *per* manufacturer’s recommendations. Multichem S Plus commercial control materials (Techno-path Manufacturing Ltd, Ballina, Ireland) intended for standard assays were analysed in two concentration levels (*1*, *3*) during the whole period of investigation. Assays and corresponding mean coefficients of variation (CV) were as follows: biuret for TP (CV = 2.5%), bromcresol green for ALB (CV = 1.9%), IFCC (International Federation of Clinical Chemistry and Laboratory Medicine) method for LD (CV = 3.2%), enzymatic for CHOL (CV = 1.9%), glycerol phosphate oxidase method for TRIG (CV = 3.0%), kinetic alkaline picrate for CREA (CV = 2.0%), the urease method for urea (CV = 2.7%), the hexokinase method for glucose (CV = 2.2%) and the 2-chloro-4-nitrophenyl-α-D-maltotrioside substrate for AMY (CV = 2.1%). The obtained CVs were within limits defined by the manufacturer’s specifications. Baseline (initial) concentration/activities (B) were considered as reference for the calculation of deviation (D) from initial value. Immediately after baseline analysis, each pleural/peritoneal fluid sample was aliquoted into secondary tubes without additive, resulting in five aliquots *per* sample. Pleural and peritoneal fluid aliquots were stored separately. Aliquots were stoppered to minimize evaporation, labelled to ease further analysis and marked with numbers from 1 to 5. Aliquots denoted as 1 were stored in an upright position at average room temperature (RT) of 24.7 (24.1 to 25.3) °C for 6 hours. Ambient temperature was measured using a calibrated thermometer (TFA Dostmann GmbH, Wertheim-Reicholzheim, Germany). Aliquots denoted from 2 to 5 were stored for 3, 7, 14 and 30 days at an average temperature of - 19.8 (- 19.4 to - 20.4) °C, respectively. At the end of each storage period, samples were thawed (if applicable) and visually checked for the presence of particulate matter. If present, particulates were recorded; samples were gently mixed and once again centrifuged (as described above). Particulates were removed and repeated measurements of all analytes were performed.

### Statistical analysis

Data were tested for normality using the Kolmogorov-Smirnov test. Data were not normally distributed and thus presented as median and interquartile range. The Friedman ANOVA test was used to test differences between baseline concentrations/activities of chemistry analytes tested and concentrations/activities measured after each storage period. Mean D was calculated for each parameter and respective storage period using the following equation: D = (X - B)/B x 100, where X represents the result obtained after respective storage period (from 1 to 5) and B represents the baseline concentration/activity. The calculated D was compared to stability limits (SL) set at 6, 7, 12, 9, 13, 9, 8, 7 and 15% for TP, ALB, LD, CHOL, TRIG, CREA, urea, glucose and AMY, respectively. Stability limits were defined according to the Croatian centre for quality assessment in laboratory medicine (CROQALM) criteria for standard (serum) samples which are based on components of biological variation and widely used in Croatia (*19*). Statistical analysis was performed using MedCalc v.11.5 (Ostend, Belgium). P < 0.05 was considered statistically significant.

## Results

The characteristic of the population studied is presented in Table 1. After visual inspection of all the 246 aliquots tested, particulate matter (*i.e.* cloudy, fibrin-like particulates) was found in only 8%.

**Table 1:** Characteristics of the subjects’ population

**Variable**	**All fluids** **(N = 41)**	**Pleural fluid** **(N = 29)**	**Peritoneal fluid** **(N = 12)**
**Males, N**	31	21	10
**Age, years**	70 (49-94)	74 (55-94)	69 (49-82)
**Location (ward)**			
Pulmology	16	14	2
Hepatology	11	4	7
Nephrology	3	2	1
Haematology	5	5	0
Cardiology	2	2	0
Emergency	2	0	2
Intensive care	2	2	0
**Effusion aetiology**			
Pneumonia	8	8	0
Liver cirrhosis	4	0	4
Malignancy	17	11	6
Cardiovascular disorders	12	10	2
Age is presented as median (range).			

Mean baseline concentrations/activities and mean concentrations/activities of the chemistry analytes tested in pleural and peritoneal fluid samples after each storage period are presented in Tables 2 and 3, respectively. The comparison of pleural fluid TP and CHOL concentrations in all the observed storage periods revealed no statistically significant difference. However, when concentrations of ALB, LD, TRIG, CREA, urea, glucose and AMY measured after storage in different conditions were compared by means of Friedman ANOVA, a statistically significant difference was found. The decrease was most prominent for LD and glucose (pairwise comparison not shown). After comparing peritoneal fluid samples from each storage period, no statistically significant differences were found for TP, ALB, TRIG, urea and AMY. Lactate dehydrogenase, CHOL, CREA and glucose concentrations were found significantly different, and again the most prominent decrease was noted for LD and glucose (pairwise comparison not shown).

**Table 2:** Concentrations and enzyme activities of analytes at baseline and after each storage period with corresponding deviations for pleural fluid samples

**Analyte, unit** **/ mean deviation from baseline**	**Baseline**	**6 hours** **at RT**	**3 days** **at - 20°C**	**7 days** **at - 20°C**	**14 days** **at - 20°C**	**30 days** **at - 20°C**	**P**	**SL, %**
**TP, g/L**	29 (26-42)	30 (26-43)	31 (27-42)	31 (27-42)	32 (27-43)	32 (27-41)	0.265	/
**TP, %**	/	0.74	0.57	0.58	0.28	0.15	/	± 6
**ALB, g/L**	17.6 (14.5-26.4)	17.5 (14.9-26.5)	17.4 (14.8-26.1)	17.1 (14.8-26.2)	17.7 (14.7-25.7)	17.3 (14.6-25.8)	0.001	/
**ALB, %**	/	0.78	0.35	0.24	- 0.72	- 0.72	/	± 7
**LD, U/L**	171 (129-649)	164 (127-635)	158 (107-382)	145 (100-379)	137 (97-376)	123 (89-344)	< 0.001	/
**LD, %**	/	- 1.52	**- 13.96**	**- 20.49**	**- 22.54**	**- 29.52**	/	± 12
**CHOL, mmol/L**	1.5 (1.0-1.8)	1.6 (1.1-1.8)	1.6 (1.1-1.8)	1.6 (1.0-1.8)	1.5 (1.0-1.8)	1.5 (1.1-1.8)	0.170	/
**CHOL, %**	/	2.30	1.20	1.50	0.01	0.61	/	± 9
**TRIG, mmol/L**	0.3 (0.2-0.5)	0.3 (0.2-0.5)	0.3 (0.2-0.5)	0.3 (0.2-0.5)	0.3 (0.2-0.5)	0.3 (0.2-0.4)	0.024	/
**TRIG, %**	/	2.34	4.17	2.26	3.10	- 3.28	/	± 13
**CREA, μmol/L**	69 (56-81)	69 (58-79)	68 (56-76)	67 (57-79)	68 (55-78)	68 (57-77)	< 0.001	/
**CREA, %**	/	0.93	- 1.52	- 0.44	- 1.60	- 1.76		± 9
**Urea, mmol/L**	6.3 (5.5-9.6)	6.6 (5.4-9.5)	6.6 (5.3-9.8)	6.6 (5.2-9.9)	6.4 (5.2-9.6)	6.5 (5.3-9.6)	0.001	/
**Urea, %**	/	0.17	- 0.31	0.18	- 0.69	- 2.46	/	± 8
**Glucose, mmol/L**	6.5 (5.4-7.5)	6.0 (4.9-7.0)	5.8 (4.8-6.8)	5.6 (4.1-6.7)	5.5 (3.7-6.5)	4.6 (3.2-6.2)	< 0.001	/
**Glucose, %**	/	- 3.35	- 6.24	**- 10.26**	**- 15.36**	**- 23.37**	/	± 7
**AMY, U/L**	30 (20-41)	30 (21-42)	30 (20-40)	30 (21-40)	30 (20-40)	30 (20-41)	< 0.001	/
**AMY, %**	/	0.79	1.26	- 0.81	- 1.29	- 0.71	/	± 15
Data is presented as median (interquartile range). TP - total proteins. ALB – albumin. LD - lactate dehydrogenase. CHOL – cholesterol. TRIG – triglycerides. CREA – creatinine. AMY – amylase. Analytes concentrations/activities are presented as median and interquartile range. Differences between storage periods were tested using the Friedman test. P < 0.05 was considered statistically significant. RT – room temperature. SL – stability limit criteria according to the Croatian centre for quality assessment in laboratory medicine (CROQALM). Deviations exceeding SL are given in bold.								

**Table 3:** Concentrations and enzyme activities of analytes at baseline and after each storage period with corresponding deviations for peritoneal fluid samples

**Analyte, unit** **/ mean deviation from baseline**	**Baseline**	**6 hours** **at RT**	**3 days** **at - 20°C**	**7 days** **at - 20°C**	**14 days** **at - 20°C**	**30 days** **at - 20°C**	**P**	**SL, %**
**TP, g/L**	21 (17-32)	21 (17-32)	21 (17-32)	21 (17-32)	21 (16-32)	21 (17-32)	0.121	/
**TP, %**	/	1.10	0	0.75	- 1.36	0.75	/	± 6
**ALB, g/L**	9.4 (7.1-19.1)	9.4 (7.0-19.0)	9.4 (7.1-19.0)	9.4 (7.2-18.7)	9.4 (6.7-18.8)	9.3 (7.3-18.7)	0.253	/
**ALB, %**	/	- 0.21	- 0.86	0.07	- 1.76	- 0.67	/	± 7
**LD, U/L**	75 (45-102)	80 (48-110)	58 (42-101)	55 (39-93)	58 (30-93)	47 (25-79)	< 0.001	/
**LD, %**	/	6.20	**- 14.94**	**- 20.56**	**- 24.10**	**- 35.29**	/	± 12
**CHOL, mmol/L**	0.8 (0.6-1.6)	0.8 (0.6-1.7)	0.8 (0.6-1.7)	0.9 (0.6-1.7)	0.8 (0.6-1.7)	0.8 (0.6-1.7)	0.036	/
**CHOL, %**	/	0.60	0.86	1.79	0.86	3.16	/	± 9
**TRIG, mmol/L**	0.4 (0.2-0.6)	0.4 (0.2-0.6)	0.4 (0.2-0.6)	0.4 (0.2-0.6)	0.4 (0.3-0.6)	0.4 (0.2-0.6)	0.076	/
**TRIG, %**	/	- 1.85	- 1.96	2.78	6.69	- 1.75	/	± 13
**CREA, μmol/L**	99 (58-151)	98 (57-150)	97 (56-149)	97 (55-155)	97 (56-151)	95 (58-148)	0.021	/
**CREA, %**	/	- 0.77	- 2.63	- 2.19	- 3.56	- 2.36	/	± 9
**Urea, mmol/L**	8.4 (5.4-13.1)	8.5 (5.4-13.3)	8.7 (5.4-13.2)	8.6 (5.1-13.2)	8.7 (5.2-13.2)	8.6 (5.5-13.2)	0.883	/
**Urea, %**	/	1.25	1.16	0.97	1.08	1.09	/	± 8
**Glucose, mmol/L**	7.4 (5.5-8.3)	7.3 (5.4-8.6)	7.0 (5.1-8.2)	6.8 (4.9-8.2)	6.5 (4.5-7.8)	5.8 (4.0-7.6)	< 0.001	/
**Glucose, %**	/	- 0.94	- 5.24	**- 7.12**	**- 10.90**	**- 17.37**	/	± 7
**AMY, U/L**	19 (12-34)	20 (34-33)	20 (12-33)	20 (12-33)	20 (12-32)	20 (12-34)	0.554	/
**AMY, %**	/	3.17	1.00	1.49	- 1.29	0.81	/	± 15
Data is presented as median (interquartile range). TP - total proteins. ALB – albumin. LD - lactate dehydrogenase. CHOL – cholesterol. TRIG – triglycerides. CREA – creatinine. AMY – amylase. Analytes concentrations/activities are presented as median and interquartile range. Differences between storage periods were tested using the Friedman test. P < 0.05 was considered statistically significant. RT – room temperature. SL – stability limit criteria according to the Croatian centre for quality assessment in laboratory medicine (CROQALM). Deviations exceeding SL are given in bold.								

Calculated mean D values with corresponding acceptable SL are presented in Tables 2 and 3. Mean D values for TP, ALB, CHOL, TRIG, CREA, urea and AMY in both pleural and peritoneal fluid samples did not exceed the predefined SL in all storage periods tested. However, a significant loss of stability was observed for LD when pleural and peritoneal fluid samples were stored for 3, 7, 14 and 30 days at - 20°C. Furthermore, a significant loss of stability in glucose for both serous fluids tested was observed when samples were stored for 7 days at - 20°C. Lactate dehydrogenase and glucose in serous fluids stored at RT were stable for 6 hours, as demonstrated by D values not exceeding the pre-set criteria.

## Discussion

Our investigation on the stability of clinically relevant chemistry analytes in pleural and peritoneal fluid samples demonstrates that, after collection in plain (no additive) tubes, transport to the laboratory and centrifugation, all the chemistry analytes tested are stable at RT for 6 hours. Furthermore, these serous samples might be stored for 30 days at - 20°C without affecting the stability of TP, ALB, CHOL, TRIG, CREA, urea and AMY concentrations/activities. This means that such samples might be subjected to long-term preservation for validation or quality control purposes of these analytes. On the contrary, the stability of LD in pleural and peritoneal fluid is compromised when such samples are stored at - 20°C. In addition, glucose in pleural and peritoneal fluids is stable only for 3 days at - 20°C. Thus, serous samples cannot be stored at - 20°C for long-term preservation of LD and glucose. The overall quality of samples after freezing assessed by visual inspection was found satisfactory. To the best of our knowledge, this is the first study investigating the stability of analytes in pleural and peritoneal effusions.

The stability of the most commonly requested tests in pleural effusions was investigated in an early work by Antonangelo *et al.* (*20*). They found that TP and albumin in pleural effusions are stable for a week regardless if stored at 21°C or - 20°C. Furthermore, in this study pleural fluid CHOL was stable for 4 days at 21°C (RT), and 14 days if frozen. Triglycerides were found stable for 4 days, independent of the storage conditions. Glucose was stable for 2 days at RT, while if frozen it maintained stability for a week. The stability of LD in pleural fluid samples was 4 days at RT, while if pleural fluid samples were stored at - 20°C, its stability was compromised as early as after 1 day. It is challenging to compare our results to those mentioned above because of differences in study design and defined stability criteria. Our results obtained comparing D to stability criteria based on components of biological variation for standard fluids, suggest that TP, ALB, CHOL, TRIG, CREA, urea and AMY are stable if pleural fluid samples are stored for 6 hours at RT (which is in agreement with the results of Antonangelo *et al.*) and for 30 days at - 20°C. As for glucose in pleural fluid samples, it might be safely stored for 6 hours at RT, or alternatively up to 3 days at - 20°C. Unlike the previously mentioned results, we found that glucose in pleural fluid samples is not stable up to a week if stored at -20°C. The results for pleural fluid LD in both investigations were found comparable: LD stability is compromised if samples are stored at - 20°C. This has been attributed to the decreased stability of isoenzymes LD4 and LD5 (*20*).

A recent analytical validation study of several body fluids investigated the stability of TP, ALB, CHOL, TRIG, CREA, urea, AMY, glucose and LD in ascites and pleural fluid samples at both 4°C and - 70°C for up to 14 days (*12*). Again, a direct comparison of stability results is difficult due to different study settings (*i.e.* different acceptability criteria, limited number of samples, RT stability not investigated). Interestingly, all the evaluated analytes were stable for at least 14 days if stored at - 70°C, including LD (*12*). We found that pleural and peritoneal fluid TP, ALB, CHOL, TRIG, CREA, urea and AMY were stable for up to 30 days if stored at - 20°C, while glucose stability in peritoneal (and pleural) fluid was preserved up to 3 days if stored frozen (*i.e.* at - 20°C). On the contrary, our results demonstrate that pleural and peritoneal fluid LD should not be stored frozen.

The most recent analytical validation of assays in body fluid analysis performed by Block *et al.* included sample stability evaluation under a limited set of storage conditions (*i.e.* frozen for 30 days, refrigerated for up to 7 days and at RT for 1 and 7 days post-collection) (*4*). Their results for TP, ALB, CREA, LD, glucose, urea and AMY stored at RT corroborate ours. However, when these analytes were stored for 30 days at - 24 to - 40°C, rather conflicting results were obtained. As observed in one previously mentioned study, the stability results for LD in freezing conditions were found comparable to ours; but according to their results, glucose was stable if stored frozen for up to 30 days post-collection, while ALB, CREA and TP were not. These results should be interpreted with caution due to a very limited number of peritoneal (N = 5) and pleural (N = 1) samples investigated and different acceptable criteria used compared to our investigation (*4*).

Our investigation includes several limitations. Due to the invasive collection procedure, only a limited number of pleural and peritoneal samples were included in our study. Furthermore, limited sample volumes did not allow the analysis of replicate measurements in each time point. The transport conditions of the samples to the laboratory are not closely monitored (*i.e.* exact collection time and transport temperature) and thus additional sources of bias cannot be excluded. Finally, our current routine laboratory workflow does not include the assessment of haemolysis, icterus and lipemia in pleural and peritoneal fluid samples, because it is assumed that these preanalytical interferences affect serous samples similarly to serum samples.

## Conclusions

We present the results of the first study dedicated specifically to the stability evaluation of clinically relevant chemistry analytes in pleural and peritoneal fluid samples. Our results demonstrated that TP, ALB, LD, CHOL, TRIG, CREA, glucose, urea and AMY in both fluids tested are stable if stored at RT for 6 hours and/or for 30 days at - 20°C However, serous samples should not be stored at - 20°C for long-term preservation of LD because of its compromised stability in such conditions. This might be applied, to an extent, to glucose in pleural and peritoneal fluid samples, whose stability is up to 3 days at - 20°C. The stability of LD and glucose in serous fluids stored at RT is limited to 6 hours. The transferability of stability data is crucial for their reliable implementation and this fact should be carefully considered in future studies in order to amend and/or confirm our results.
